# Understanding the Structure–Activity Relationship through Density Functional Theory: A Simple Method Predicts Relative Binding Free Energies of Metalloenzyme Fragment-like Inhibitors

**DOI:** 10.1021/acsomega.2c08156

**Published:** 2023-06-06

**Authors:** Silvana Vasile, Katarina Roos

**Affiliations:** Department of Cell and Molecular Biology, Uppsala University, Uppsala 751 23, Sweden

## Abstract

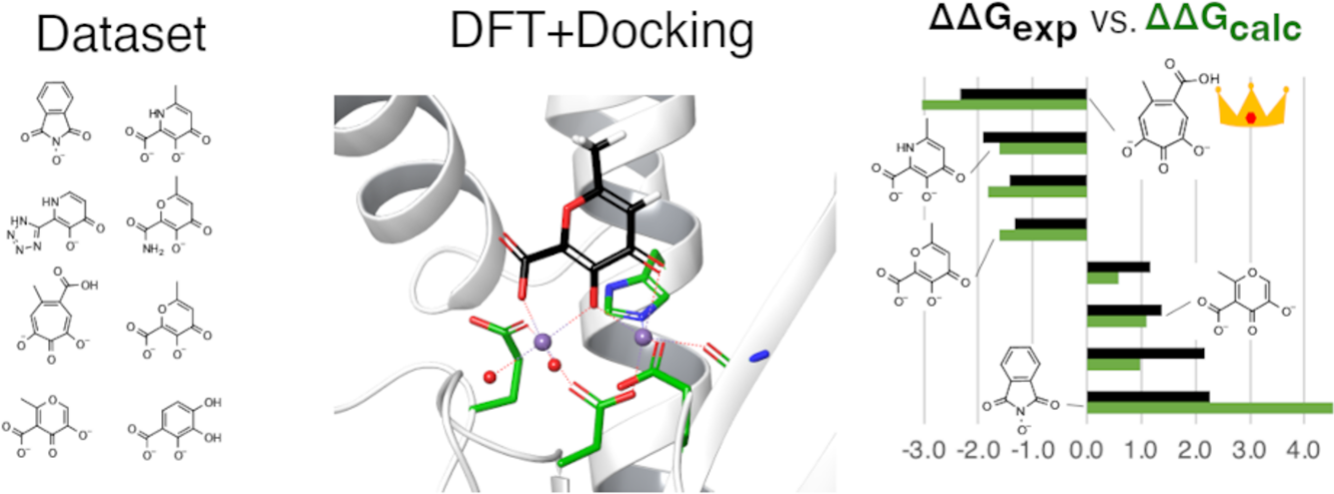

Despite being involved in several human diseases, metalloenzymes are targeted by a small percentage of FDA-approved drugs. Development of novel and efficient inhibitors is required, as the chemical space of metal binding groups (MBGs) is currently limited to four main classes. The use of computational chemistry methods in drug discovery has gained momentum thanks to accurate estimates of binding modes and binding free energies of ligands to receptors. However, exact predictions of binding free energies in metalloenzymes are challenging due to the occurrence of nonclassical phenomena and interactions that common force field-based methods are unable to correctly describe. In this regard, we applied density functional theory (DFT) to predict the binding free energies and to understand the structure–activity relationship of metalloenzyme fragment-like inhibitors. We tested this method on a set of small-molecule inhibitors with different electronic properties and coordinating two Mn^2+^ ions in the binding site of the influenza RNA polymerase PA_N_ endonuclease. We modeled the binding site using only atoms from the first coordination shell, hence reducing the computational cost. Thanks to the explicit treatment of electrons by DFT, we highlighted the main contributions to the binding free energies and the electronic features differentiating strong and weak inhibitors, achieving good qualitative correlation with the experimentally determined affinities. By introducing automated docking, we explored alternative ways to coordinate the metal centers and we identified 70% of the highest affinity inhibitors. This methodology provides a fast and predictive tool for the identification of key features of metalloenzyme MBGs, which can be useful for the design of new and efficient drugs targeting these ubiquitous proteins.

## Introduction

Around one third of proteins contain metal ion cofactors, which play a structural or functional role. The function of the metal ions present in the active sites of metalloenzymes is to catalyze chemical reactions. Metalloenzymes are biologically ubiquitous and constitute interesting targets in drug discovery due to their involvement in a wide range of human diseases, such as cancer,^1,2^ cardiovascular diseases,^3,4^ neurodegenerative disorders,^4,5^ and viral and bacterial infections.^6−11^

Metalloenzyme activity can be abolished by inhibitors binding to the metal ion(s) in the active site, thus preventing metal-mediated catalysis. The typically large bond enthalpy associated with the coordination of inhibitors to the metal sites makes the dative bonds strong and the binding process reversible at the same time.^12^ Nonetheless, only a small percentage of recently FDA-approved drugs inhibit metalloenzymes.^13^ Metalloenzyme inhibitors often consist of a metal binding group (MBG) that coordinates the active site metal ion(s) and is connected to the drug-like part of the compound (backbone) through a linker. The backbones interact with the protein binding sites through hydrogen bonds and non-polar interactions and their variety reflects the heterogeneity of the active sites.^14^ In contrast, the exploration of the chemical space for MBGs has been limited, as only four classes (hydroxamic acids, thiols, carboxylic acids, and phosphonates/phosphinates) are principally used at present.^15^ This is a limiting factor to the development and the optimization of new and more potent drugs, considering the poor pharmacokinetic properties of hydroxamic acids^2,16−18^ and thiols.^19,20^ More recently, the use of libraries of fragment-like compounds designed to bind to metal centers in fragment-based drug discovery (FBDD) was introduced to overcome the lack of chemical diversity among MBGs and their poor optimization.^21^ The exploration of the binding site is more effective when fragments are used compared to sterically hindered large molecules,^22^ and in general this approach leads to good ligand efficiencies.^23^

The use of computational methods in drug discovery has expanded in the past decades thanks to the increasing computational power and the availability of computational tools.^24−26^ One significant goal in computational drug discovery is to predict the binding affinity of compounds to macromolecules (enzymes and proteins) with “chemical accuracy” (∼ ±1 kcal/mol), for which a variety of methods have been developed and optimized. Approaches based on energy functions, such as docking, are able to predict protein–ligand complex geometries with precision and are very fast at scoring the binding affinities but they lack accuracy in this regard.^27^ Recently, a docking strategy to predict metalloenzyme–MBG interactions was presented, which can reproduce the binding modes of metalloenzyme inhibitors with different MBGs starting from DFT-optimized MBG conformations.^28^ Another popular methodology is to couple molecular dynamics (MD) or Monte Carlo (MC) simulations to free energy perturbation (FEP) simulations: this approach employs statistical mechanics to estimate binding affinities from MD (or MC) sampling of interaction energies.^24^ These are powerful and very accurate methods that, in principle, can estimate absolute binding free energies^29^ but have a higher computational cost compared to docking. Moreover, convergency problems arise when the ends of the transformation are too chemically different^30^ but dual topology methods^31^ and progress in single topology approaches can solve this aspect.^32^ Additionally, due to the non-optimal description of nonclassical phenomena such as charge transfer, polarization, and π–π interactions,^33−36^ force field-based methods are limited in the treatment of systems where the contribution of these effects is great, in particular in metalloenzymes.^37^ On the other hand, the explicit treatment of electrons by quantum-mechanical (QM) methods may improve the accuracy of ligand binding predictions. This advantage may overcome the relatively high computational cost usually associated with QM calculations, which has progressively reduced thanks to the expanding computer power.^25,38,39^ Different QM methods can be applied to calculate binding affinities, from semi-empirical QM calculations and density functional theory (DFT) to coupled-cluster methods.^25^ Although in general too computationally demanding to describe the dynamics of biological systems, they are applicable to systems with available crystal structures and where strong key interactions between ligand and receptor are the main contributing factor to the binding energies, such as in the case of metalloenzymes. In a study of 2014, a DFT-based model was developed to rank the potencies of a series of drug scaffolds with the aim of prioritizing the most potent cores for a structure-based drug discovery project, using a simplified model of the binding site including only key residues.^40^ Previously, a similar DFT-based approach (QM cluster) has been successfully applied to model complex enzymatic reactions.^41,42^ We herein expanded and adapted this method to predict the relative binding free energies of a series of small-molecule compounds with a common metal-binding scaffold that inhibits the influenza RNA polymerase PA_N_ endonuclease.^12^ The explicit treatment of electrons provided by DFT could clarify the actual contribution of metal coordination to binding free energies and provided insights on the relevance of parts of the binding site possibly overlooked in the experimental design of the inhibitors. By enabling the exploration of plausible binding poses with automated docking, in lack of crystallography data, we aimed to develop a general computational method to predict binding affinities: the protocol presented here may contribute to the search for new and more efficient MBGs and to the design of novel metalloenzyme inhibitors. Thanks to the small number of atoms necessary to represent the binding site, this approach could constitute an accessible tool to be used in the early stages of a drug discovery project with limited computational cost.

## The Target: PA_N_ Endonuclease

The influenza virus is the cause of seasonal epidemics that are linked to considerable deaths, especially among the elderly and individuals with high-risk factors. Although influenza infections usually cause mild symptoms, secondary opportunistic infections may occur and ultimately lead to complications and death.^13^ Influenza vaccines protect against the infection and can reduce the severity of the symptoms but they must be administered each year due to rapidly emerging virus strains and mutations.^43^ The effectiveness of the currently available antivirals is also affected by the rapid mutation of the virus and its antigenic variation,^44−46^ and therefore, the development of new inhibitors to fight influenza infections is still actual.

The polymerase PA endonuclease is a member of the influenza RNA polymerase complex. This highly conserved domain regulates the lifecycle of the virus, as it generates primers for the synthesis of the viral mRNA through “cap-snatching”.^47^ Its catalytic site is located in the N-terminus,^47,48^ where two divalent cations are coordinated by a histidine (His41), an isoleucine (Ile120), and three acidic residues (Asp108, Glu80, and Glu119).^49,50^ A water molecule acts as nucleophile when the phosphodiester backbone of the host mRNA is hydrolyzed by the endonuclease, while the coordination with the two metal ions stabilizes the transition state intermediate.^51−55^ The nature of the catalytic ions has been extensively discussed, as both Mn and Mg have been found through X-ray crystallography,^47,48,51,52,56−58^ but the presence of two Mn^2+^ has been shown to enhance the activity of the endonuclease.^59,60^

Small-molecule inhibitors suppress the endonuclease activity by binding to the metal cations.^21,52,53,55^ In particular, the majority of PA_N_ endonuclease inhibitors coordinate the metal ions through two oxygen atoms.^13^ In October 2018, the first PA_N_ endonuclease inhibitor, baloxavir marboxil, was approved by FDA but it was shown to be less effective toward influenza A viruses bearing the I38T mutation,^61^ and thus it is crucial to develop highly active and selective PA_N_ endonuclease inhibitors focusing on the MBG electronic effects. Mutations of residues coordinating the metal ions cause complete loss of viral transcription,^59^ hence disfavor substrate binding and catalytic activity. The optimal coordination of the metal cations is pivotal for the virus lifecycle: inhibitors designed to strongly coordinate the metal cations should reduce the possibility of developing antiviral resistance.^62^ Many MBGs have been explored but hydroxypyridinone was found to be the most active scaffold.^13^

In this study, we expanded a previously developed DFT-based method for predicting relative binding free energies and applied it to a set of small-molecule compounds that bind to PA_N_ endonuclease by coordinating the two metal cations through a suitable donor triad (Table 1). All the optimized structures described in this study are available in the Supporting Information.

**Table 1:**
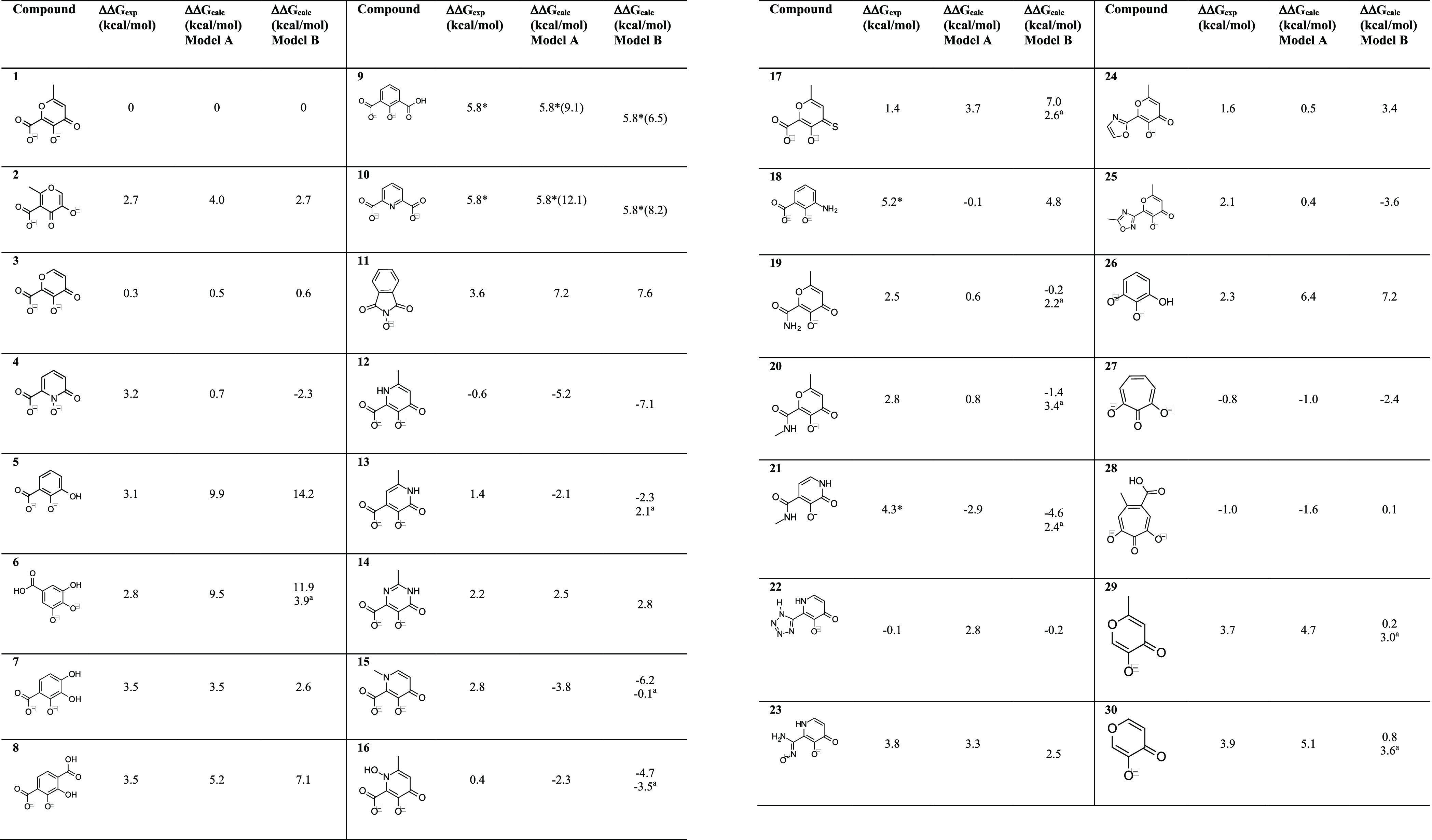
Structures of the PA_N_ Endonuclease Inhibitors of This Study and Their Relative Binding Free Energies

a Calculated relative binding free energy derived from an automated docking binding pose.

b Threshold values, with calculated values in parentheses.

## The Dataset

We extracted the dataset in Table 1 from a study of 2018,^12^ where the optimization of activity and selectivity of PA_N_ endonuclease inhibitor fragments was achieved by tuning the MBG electronic effects. We converted the pIC50 values reported in the study to Δ*G* (kcal/mol) and calculated the relative binding free energies ΔΔ*G* (kcal/mol) using the Δ*G* of compound **1** as a reference.

**Figure 1:**
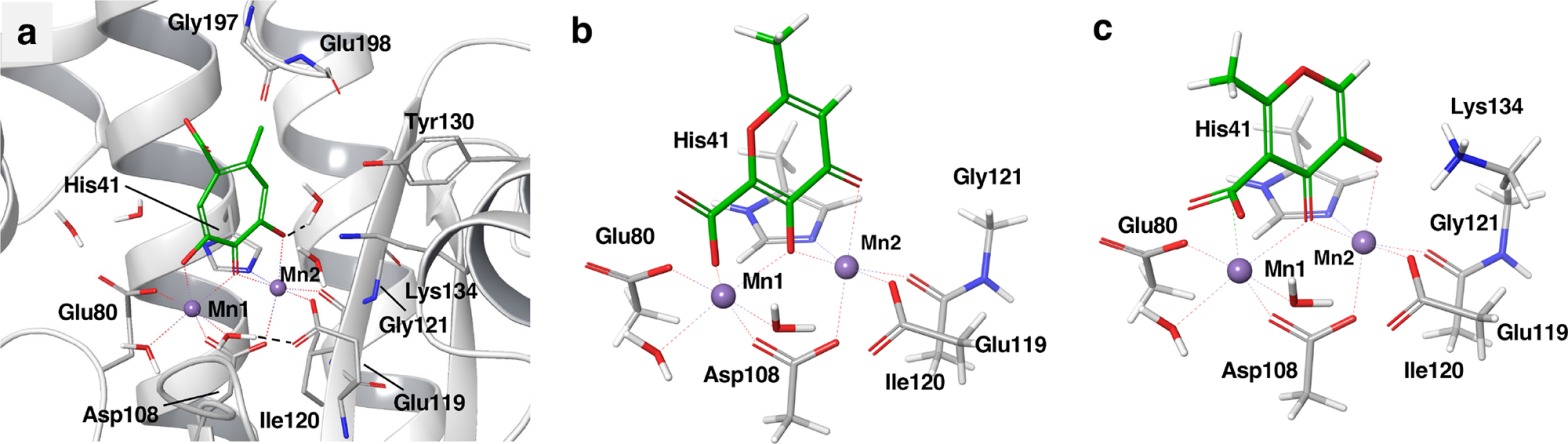
(a) Crystal structure of the complex between PA_N_ endonuclease and compound **28** (green sticks; PDB code: 6E0Q). The residues of the binding site are depicted as gray sticks, and the hydrogen bonds with water molecules are represented by black dashed lines; (b) model A: model of the binding site of PA_N_ endonuclease including only the residues coordinating the Mn ions (violet spheres), in complex with compound **1** (green sticks); (c) model B: model of the binding site of PA_N_ endonuclease including the residues coordinating the Mn ions (violet spheres) and the sidechain of Lys134, in complex with compound **2** (green sticks).

In the original study, an MBG-FBDD approach was used to optimize the activity and selectivity of fragments binding to PA_N_ endonuclease. Probing characteristics such as donor atom identity, Lewis basicity, and isosteric replacement of the coordinating groups through biochemical assays, Credille et al. found that the most active PA_N_ endonuclease inhibitors share a common donor triad. Moreover, they validated the predicted coordination of the two Mn^2+^ of the binding site through a shared triad of oxygen donor atoms with X-ray crystallography. According to their observations, the major contribution to the binding enthalpy comes from the simultaneous octahedral coordination of the two metal ions and their study emphasized the importance of the electronic characteristics of the ligand for its inhibitory activity. In particular, the greater the ligand basicity is, the stronger the interactions with the hard Lewis acidic metal centers of PA_N_ endonuclease are. Additionally, the aromaticity of the compounds and the identity of endocyclic heteroatoms influence the electronic density of the atoms involved in the metal coordination. The overall geometry of the complex is also a determining factor for the inhibitory activity of the compounds: the resemblance to the geometry of the transition state intermediate (a five-membered phosphate ester) may lead to enhanced inhibition, as shown by the binding mode (Figure 1a) and the affinity of compound **28** for the receptor (Table 1).^12^

The electronic features and the size of the compounds in Table 1 make it an optimal dataset to test and develop a DFT method that aims to predict the binding free energies, to investigate the various contributions of the metal center(s) of metalloenzymes and other residues facing the binding pocket, and identify the features that discriminate between strong and weak inhibitors.

## Results and Discussion

### Ligand Conformation and Protonation State

As shown by the crystal structures of PA_N_ endonuclease in complex with **1** and **3**,^12^ the carboxylate group of the ligands coordinates Mn1 (Figure 1b) and is coplanar to the aromatic ring, while the electronic density of the same moiety of **2** is diffuse over the ion, with the group perpendicular to the aromatic ring (Figure 1c). The lower affinity of **2**, compared to the structurally similar **1** and **3**, was associated with the non-optimal coordination between the carboxylate and Mn1, resulting in a weaker interaction with the protein.^12^ Hence, we tested the two conformers as starting points for the geometry optimization of both bound and free states of all the ligands.

Table 1 shows the protonation state used for each inhibitor in the dataset. As an approximation in our models for estimating the relative binding energies, we kept the ligand donor atom bridging the two Mn ions deprotonated both in the bound and unbound states, considering that a hard Lewis base is preferred in this position in order to form strong interactions with the metals. We report the p*K*_a_ of titratable groups in water in the Supporting Information (Table S2). In addition, we kept the carboxylate groups that do not coordinate the metal ions protonated to avoid modeling artifacts due to an excessive charge of the system.

### Binding Site Models

We built two models of the binding site, initially including only the two Mn^2+^ ions, the sidechains, and the water molecules directly coordinating the metals (model A, Figure 1b). The second model of the binding site (model B, Figure 1c) additionally included the sidechain of Lys134, possibly involved in polar interactions with the inhibitors. Larger models of the binding site (C–F) in complex with the co-crystallized inhibitors **1**–**3** and **28** were investigated. Representations of these larger models and the results obtained are included in the Supporting Information (Figure S2 and Table S5). The optimized geometries for all models in complex with the inhibitors, as well as the free ligands, are available in the Supporting Information.

To determine the relative spin of the open-shell Mn^2+^ ions in the ground state, we calculated the energies for possible combinations of spins using model B (Table S1). We adopted the spin combination (5, −5) throughout the study because its energy resulted to be the lowest.

### Binding Free Energy Calculations

We aligned all the compounds of the dataset (Table 1) to model A (Figure 1b) using the binding modes of **1**, **2**, **3**, and **28**, extracted from the respective X-ray structures, as reference, and tested both conformers of the carboxylate group (or its isostere) that coordinates Mn1 (Figure 1). We then optimized the structures of the compounds in their bound and free states in gas phase and evaluated the energies of both states by SPE calculations in solution. The binding site of PA_N_ endonuclease is solvent-exposed, therefore we considered using water as solvent as the best way to reproduce the in vivo conditions and to mimic the binding process. After selecting the conformers with the lowest energy, we calculated the binding free energy as the difference between the energy of the bound state and the energy of the free state.

### Assessing the Binding Free Energy Contribution from Metal Coordination

We used model A (Figure 1b) to assess the relative binding free energy contribution of the direct coordination of the metal ions by the fragments in Table 1. We paid special attention to the selection of the conformers for compound **1**, as its binding free energy was taken as reference for deriving the ΔΔ*G* of the other molecules. In the free state, the conformer with COO^–^ coplanar to the ring showed the lowest energy, similarly to the binding mode reported in the crystal structure. In the bound state, the inhibitor formed a hydrogen bond with one of the two waters coordinating the metals. Compound **3** showed a similar behavior, resulting in a calculated relative binding free energy of 0.5 kcal/mol, very close to the experimental value of 0.3 kcal/mol. The optimized bound state of compound **2** with the lowest energy showed the COO^–^ group perpendicular to the plane of the aromatic ring, in agreement with the crystal structure geometry, resulting in ΔΔ*G*_calc_ = 4.0 kcal/mol (ΔΔ*G*_exp_ = 2.7 kcal/mol).

The calculated relative binding free energy of the next co-crystallized inhibitor, compound **28** (a hydroxytropolone derivative showing the highest affinity in the dataset for PA_N_ endonuclease), was −1.6 kcal/mol, very close to the experimental value of −1 kcal/mol (Table 1). We achieved a similarly optimal correlation (ΔΔ*G*_calc_ = −1 kcal/mol vs ΔΔ*G*_exp_ = −0.8 kcal/mol) for compound **27**, which we assumed would bind as its derivative **28** due to their structural resemblance.

Additionally, the relative binding free energies calculated using model A showed good qualitative correlation with the experiments also for some weak inhibitors. For example, compound **11** is a very weak inhibitor that should bind in a similar manner as **28** but with less ability to resemble the transition state due to the five-membered ring geometry not allowing the optimal coordination of the Mn ions. Its lower relative affinity was qualitatively reflected by our calculations (Table 1).

Overall, the calculated binding free energies were in qualitative agreement with the experimental values for a majority of the molecules, with 21 compounds out of 30 displaying calculated ΔΔ*G*s within ±3 kcal/mol of the experimental values (Table 1). This binding site model allowed us to distinguish between weak and strong inhibitors.

In light of the results obtained with model A, the hypothesis that the greatest contribution to the binding energy of these molecules comes from the coordination of the Mn ions seemed to hold for the best inhibitors of this series, e.g., the co-crystallized compounds, but would not be enough to discriminate between weaker inhibitors of PA_N_ endonuclease. Therefore, we expanded the model to include the sidechain of Lys134, placed in the vicinity of the ligands, according to the X-ray structures, possibly forming hydrogen bonds with one of the atoms of the ligand donor triad (Figure 1b, model B).

### Tuning the Binding Free Energy by Interaction with a First-Shell Residue

The careful selection of the conformers for compound **1** proceeded as described above, and the calculated binding free energy for compound **3** was comparable to the value obtained with model A (Table 1). Perfect correlation with the experimental binding free energy was achieved for compound **2**, indicating that the hydrogen bond between Lys134 and the deprotonated hydroxyl of **2** contributes more than the same interaction with the carbonyl oxygen of **3** to the binding free energy of these two inhibitors. Additionally, with **2** being a weaker inhibitor than **1** and **3**, the interaction with Lys134 partly compensates for a weaker binding to the metal ions.

**Figure 2:**
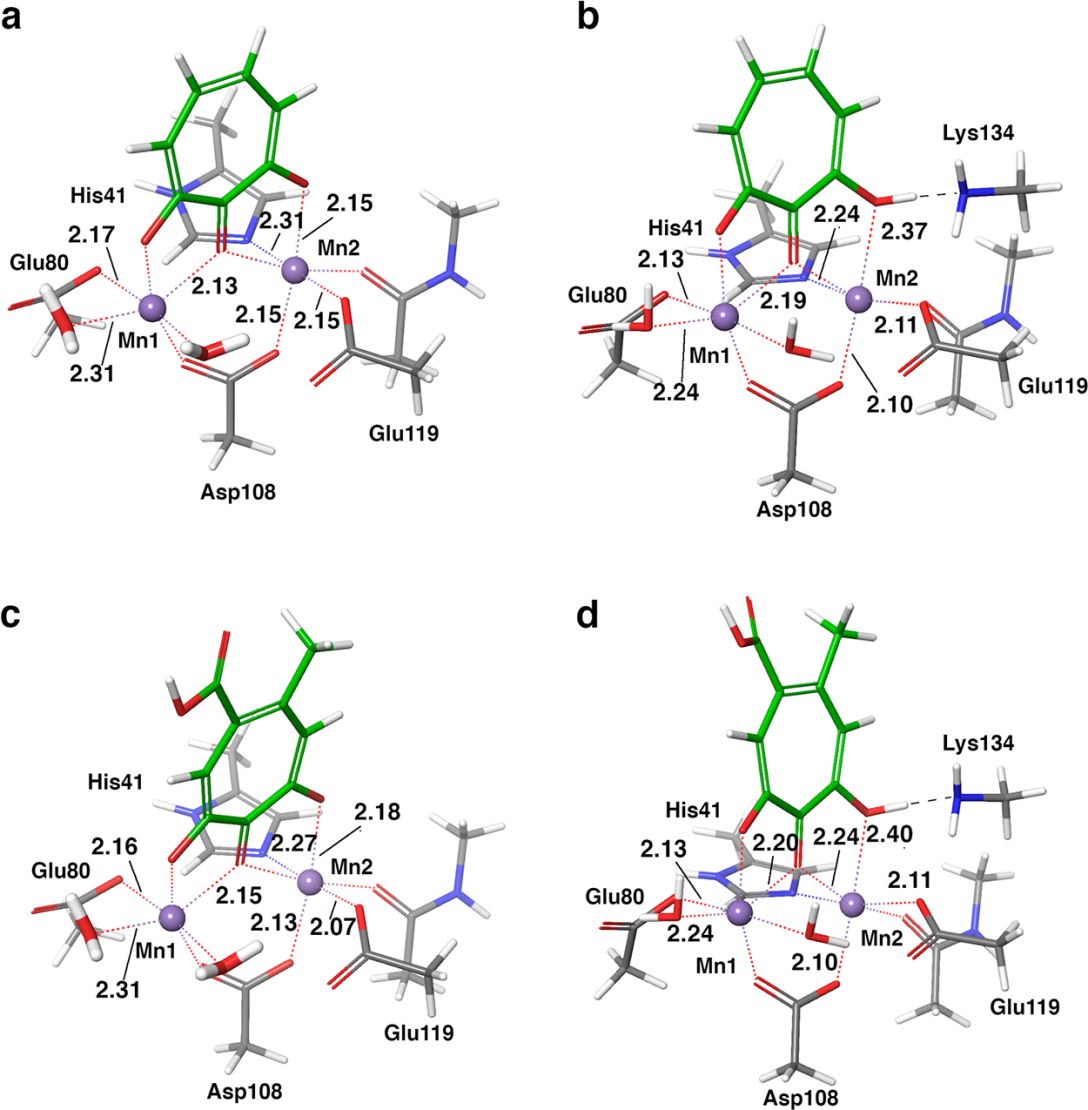
Coordination of the Mn ions by compounds **27** and **28** in model A (a, c) and model B (b, d). Only the interatomic distances that changed the most are highlighted. The proton transfers observed in model B are depicted as dashed lines between the neutral Lys134 sidechain and the hydroxyl groups of the ligands.

The bound state model of compound **28** was affected by a proton transfer from Lys134 and at the same time the prediction of the relative binding free energy raised from −1.6 kcal/mol with model A to 0.1 kcal/mol with model B, a value still close to the experimental binding free energy (−1 kcal/mol). Optimization of the bound state in water did not show the occurrence of the proton transfer, which we interpreted as an artifact caused by the optimization carried out in gas phase. Similarly, the prediction of affinity for compound **27** changed from −1 kcal/mol with model A to −2.4 kcal/mol with model B. By introducing the sidechain of Lys134 in the model of the binding site, the oxygen atoms of **27** and **28** coordinating Mn2 were both displaced by 0.22 Å compared to model A (Figure 2). The reduced accuracy in the prediction of the relative binding free energies for the best inhibitors of the series seems connected to a sub-optimal coordination of the Mn2 ion due to the potential artifact proton transfer. However, the values were still in qualitative agreement with the experiments.

As with model A, we observed an optimal correlation for compound **14** (ΔΔ*G*_calcA_ = 2.5 kcal/mol, ΔΔ*G*_calcB_ = 2.8 kcal/mol, and ΔΔ*G*_exp_ = 2.2 kcal/mol) but in contrast to model A, the conformers of the two states differed greatly: the dihedral angle between the carboxylate group of the ligand and its ring plane was 89° in the free state vs 1° in the bound state. The energetic cost associated with the different conformations in the two states is included in the model and contributes to the accurate prediction of the binding energy.

Compared to model A, the agreement between experimental and calculated relative binding free energies worsened for the manually docked poses of the inhibitors in the extended model of the binding site, model B. More specifically, only in three cases (**2**, **18** and **22**) the correlation with the experiments was recovered, while the predicted binding free energy of two compounds completely lacking the carboxylate moiety (**11** and **26**), bearing an *N*-hydroxy amidine (**23**) or an oxazole ring in its stance (**24**) did not show substantial changes compared to model A. For other compounds that replace the carboxylate group with an amide or with an oxazole derivative, the correlation with the experimental binding free energies was sensibly reduced compared to model A (**21** and **27**–**30**) or lost (**19**, **20** and **25**) (Table 1). The reduced correlation indicates that certain aspects of the modeling needed further investigation.

### Understanding SAR by Exploring Alternative Binding Modes

**Figure 3:**
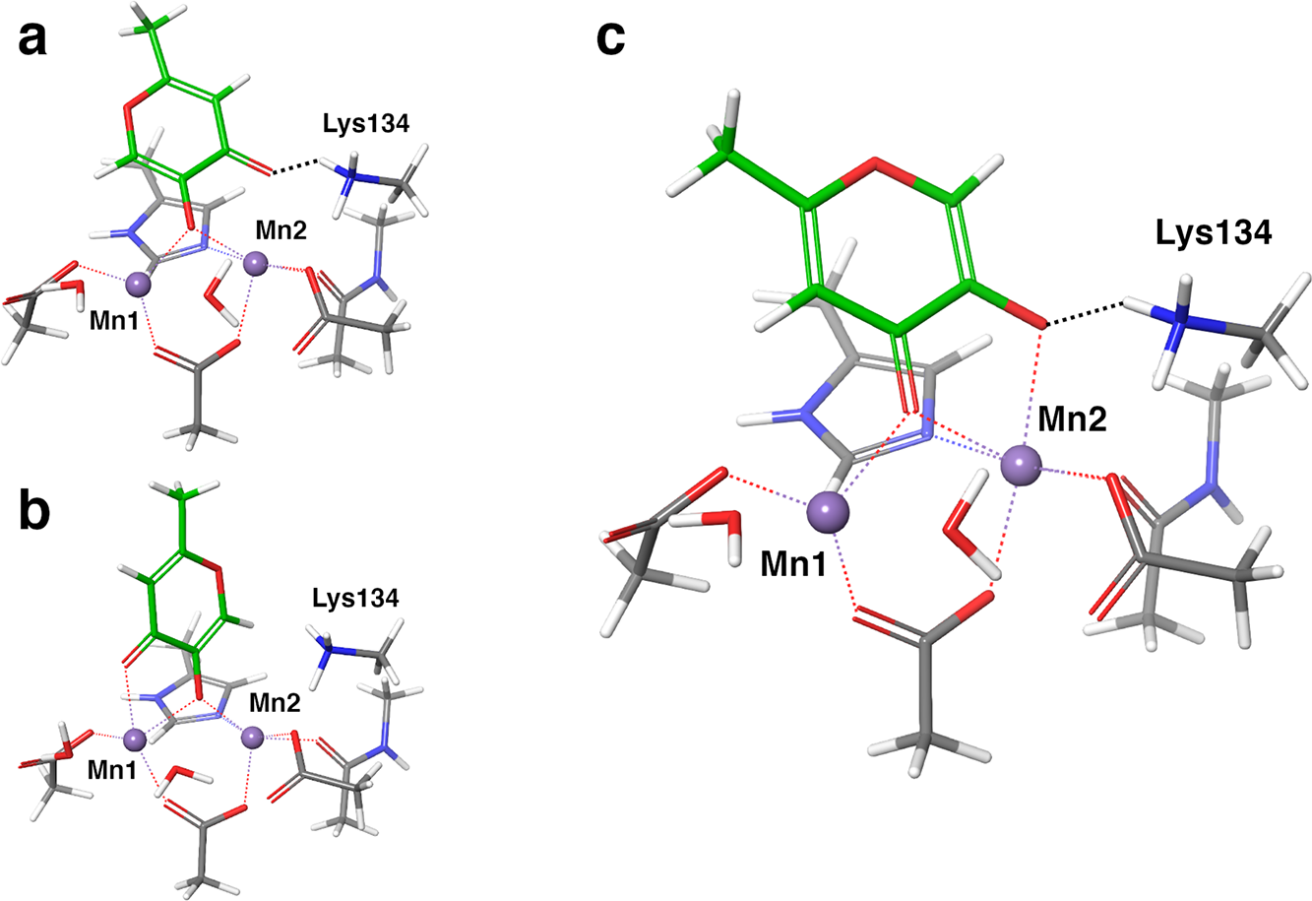
Three plausible binding poses for **29**. (a) Binding pose based on the crystal poses of **1** and **3**. A hydrogen bond between the carbonyl oxygen of **29** and Lys134 is present. (b) Binding pose based on the crystal poses of **1** and **3** rotated by 180°. No hydrogen bond with Lys134 is present, and the carbonyl oxygen of **29** coordinates Mn1. (c) Binding pose based on the crystal pose of **2**. Lys134 forms a hydrogen bond with the phenyl oxygen of **29**.

We investigated the reduced correlation observed for **29** and **30** (parent compounds of **1**–**2** and **3**, respectively) upon introduction of Lys134 in the binding site model. Both molecules are weaker inhibitors compared to **1** and **3**, with ΔΔ*G*s of 3.7 and 3.9 kcal/mol, respectively. Due to the absence of the carboxylate group in **29** and **30**, there are multiple ways to guess their binding pose by superimposition to the co-crystallized ligands **1**, **2**, and **3**. Using the pose of **1** and **3** in model B as reference, hence coordinating both the Mn ions with the phenyl oxygen and allowing their carbonyls to form a hydrogen bond with Lys134 (Figure 3a), we obtained values of ΔΔ*G*_calc_ very close to each other (as in the experiments) but also to the binding energies of **1** and **3** (Table 1). The values obtained with model A, instead, were closer to the experimental affinities (4.7 kcal/mol for **29** and 5.1 kcal/mol for **30**), indicating a weaker coordination established with the metal centers. With a rotation of 180° on the phenyl oxygen axis, we eliminated the contribution of the hydrogen bond with Lys134 in model B (Figure 3b) and the values of the calculated relative binding free energy increased greatly (ΔΔ*G*_calc_ = 11.3 kcal/mol for **29** and ΔΔ*G*_calc_ = 11.5 kcal/mol for **30**), indicating that a hydrogen bond with Lys134 compensates for the weak interaction of low-affinity inhibitors with the metal ions of the binding site of PA_N_ endonuclease. Finally, by superimposing the structures of both **29** and **30** to the crystal pose of **2**, thus restoring the hydrogen bond with Lys134 (this time with the phenolic oxygen) and using a softer Lewis base, the carbonyl oxygen, to coordinate the two Mn ions (Figure 3c), we obtained ΔΔ*G*s of 3.0 and 3.6 kcal/mol, respectively, very close to the experiments. Therefore, we concluded that weaker inhibitors (such as **2**, **29**, and **30**) may coordinate the two Mn ions through non-optimal donor atoms. The same binding pose tested on model A poorly correlated with the experimental binding free energy (Table S3), adding evidence on the importance of the hydrogen bond with Lys134 for balancing the weak binding of poor inhibitors to the metal centers.

The dataset in Table 1 includes other poor inhibitors, which can coordinate the two metal centers in different ways using Lewis bases with different strengths. For example, using the three phenolic oxygens of compound **6** as MBG, we obtained exaggerated predictions for the binding free energies (ΔΔ*G*_calc_ = 9.5 kcal/mol in model A and 11.9 kcal/mol in model B, against ΔΔ*G*_exp_ = 2.8 kcal/mol). By using a softer Lewis base to bridge the Mn ions (one of the phenolic oxygens in meta-position to COO^–^), we obtained a value of ΔΔ*G*_calc_ = 3.9 kcal/mol in model B, within the chemical accuracy of ΔΔG_exp_, while the prediction remained similar for model A (Table S3). Again, modulating the strength of the Lewis base bridging the metals and establishing a hydrogen bond between compound **6** and Lys134 resulted in an accurate prediction of its affinity for PA_N_ endonuclease.

Testing alternative binding poses for compounds that lack one atom of the donor triad or that can coordinate the Mn ions in different ways improved the prediction of experimental binding free energies. Hence, we introduced automated docking to test the effect of alternative binding poses on the performance of our DFT-based method.

### Combining DFT with Automated Docking

We ran automated docking with GOLD on the same set of molecules to obtain alternative plausible binding poses. We tested all the docking poses that interacted with the Mn^2+^ ions and with Lys134 and that did not imply extensive non-polar interactions between the ligand and the receptor at the same time, since we focused on probing the interactions with the metal centers and the first-shell residues. The new binding modes of **6**, **29**, and **30** described in the previous section were independently found through docking.

**Figure 4:**
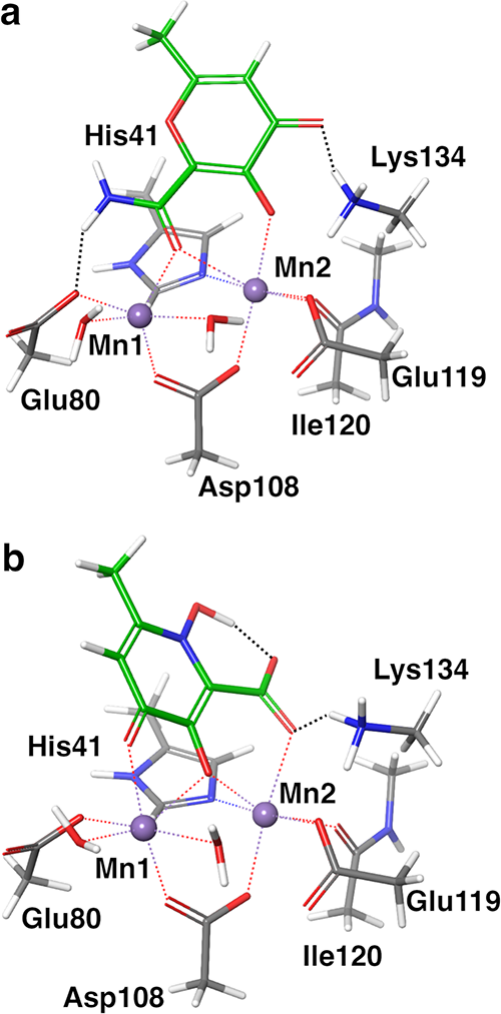
(a) Binding pose that tests the influence of the strength of the Lewis base coordinating both metal centers (optimized bound state of compound **19**); (b) optimized structure of the bound state of compound **16** interacting with Lys134 through the carboxylate group. The internal hydrogen bond of **16** and the hydrogen bonds between the ligands and the binding site are depicted as black dotted lines.

The correlation for compounds **19**–**21**, all with an amide group as carboxylate isostere, benefitted greatly from the alternative binding pose depicted in Figure 4a (Table 1). Both the non-optimal coordination of Mn^2+^ and the weaker Lewis-base character of the bridging atom compared to a phenolic oxygen contributed to accurate reproduction of the relative binding energies of these compounds. Moreover, the loss of correlation observed when the alternative binding pose was tested with model A (Table S3) corroborates the hypothesis that a hydrogen bond with Lys134 compensates for the weaker interactions between these ligands and the metal centers. In the absence of specific structural information for these inhibitors, we do not exclude the fact that the binding mode of these molecules actually deviates from crystal structures of **1**, **2**, and **3** and may resemble the one in Figure 4a.

A similar arrangement of compounds **13** and **17** in the binding site of PA_N_ endonuclease improved the correlation with the experiments: as a matter of fact, the ΔΔ*G*_calc_ obtained using this alternative binding mode reflected the similar binding free energy of the two inhibitors (respectively, ΔΔ*G*_calc_ = 2.1 kcal/mol and ΔΔ*G*_calc_ = 2.6 kcal/mol vs ΔΔ*G*_exp_ = 1.4 kcal/mol for both **13** and **17**).

Compound **16**, with experimental ΔΔ*G* similar to **3**, presents an internal hydrogen bond (Figure 4b). Both model A and model B failed to predict its relative binding free energy (Table 1), therefore we tested the effect of changing the identity of the atom involved in the hydrogen bond with Lys134 by flipping the molecule around the axis of the phenolic C–O bond, as in Figure 4b. This binding mode was not found via automated docking, which indicated instead the coordination of the two metal ions through the carboxylate moiety as the most energetically favored (Figure S1). It involves non-polar interactions with residues outside the first shell, hence it was not tested. The alternative binding pose in Figure 4b might mitigate the contribution of the internal hydrogen bond to the binding free energy. Although we actually observed such mitigation, as ΔΔ*G*_calc_ passed from −4.7 to −3.5 kcal/mol (ΔΔ*G*_calc_ = −2.5 kcal/mol with model A), we are still far from the experimental ΔΔ*G* of 0.4 kcal/mol. The ΔΔ*G*_calc_ obtained testing this binding pose on model A (−0.8 kcal/mol) showed improved correlation with the experimental binding free energy of **16**. We believe that the internal hydrogen bond contributes to the underestimation of the relative binding free energy of **16** and that an explicit treatment of the solvent may reduce this effect.

### Performance of the Method

**Figure 5:**
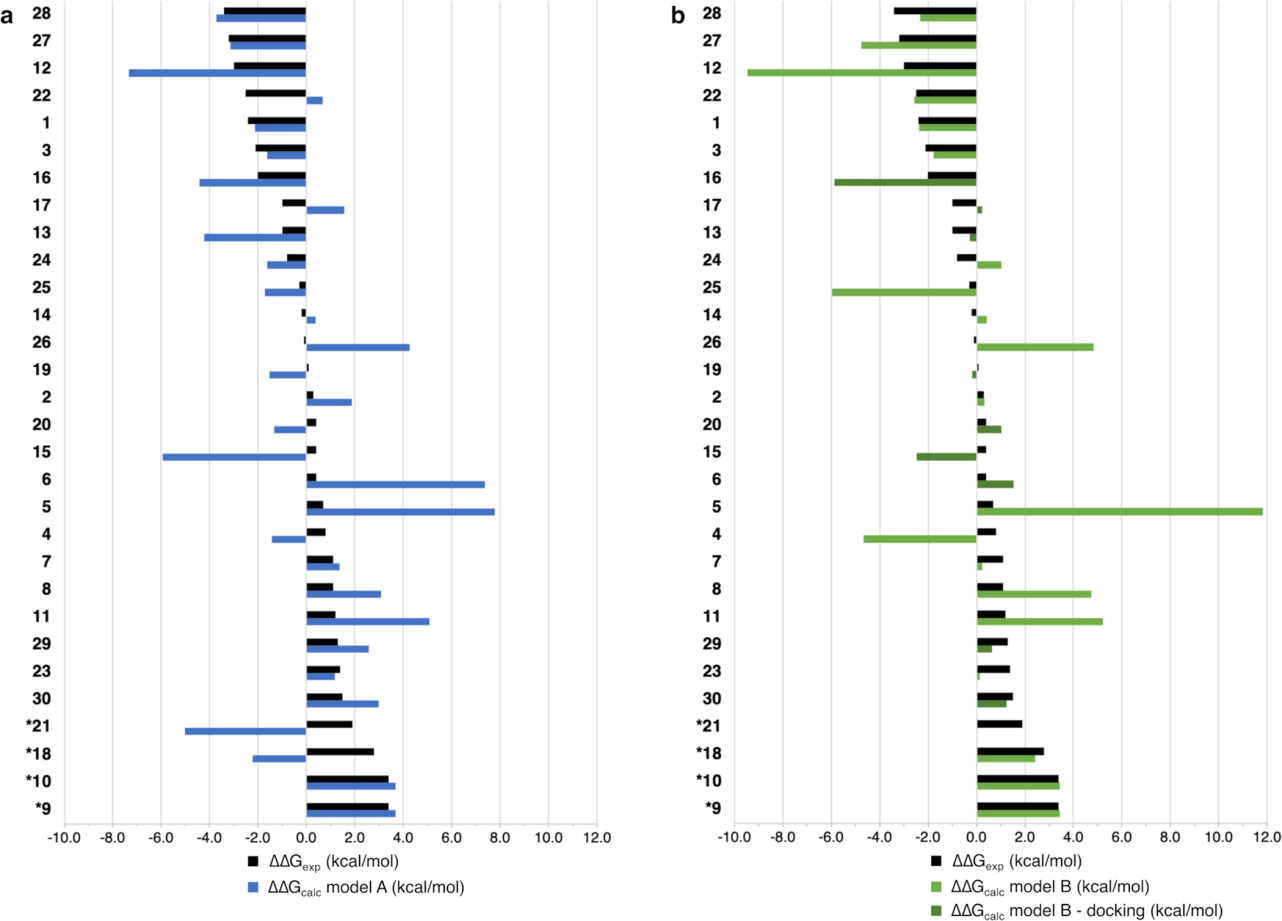
Comparison of the results obtained with (a) model A and (b) model B with the experimental binding free energies. The histograms report the ΔΔ*G* of each compound normalized by the averages of the relative binding energies (with compound **1** as reference) and sorted by descending experimental affinity. The ΔΔ*G*_calc_ values of the compounds indicated with an asterisk (*) were approximated to the threshold values of the experiments when they exceeded them.

Figure 5 shows how reliable our computational method is in distinguishing between strong and weak inhibitors and how the performance varies using different models of the binding site. In particular, the correlation between calculated and experimental binding free energies improves for some of the weaker inhibitors by expanding the model of the binding site and introducing docking poses. We calculated the average relative binding free energy for each group (experimental, model A, model B including the ΔΔ*G* obtained from docking poses) and used these averages to normalize all the ΔΔ*G* values of the respective group. In this way, we could directly compare the performances of each model with the experiments.

As mentioned above, a minimal model of the binding site, including only the first coordinating shell of the metal ions, worked very well for the most potent inhibitors (namely, **1**, **3**, **12**, **16**, **22**, **27**, and **28**). The only instance where model B performed better than model A was compound **22**, which replaces the carboxylate group with a bulky acidic tetrazole, possibly distorting the coordination geometry of the Mn ions. In particular, the correlation with the experiment was completely recovered after establishing a hydrogen bond with the sidechain of Lys134.

In the previous sections, we discussed how the introduction of an additional polar sidechain in the model of the binding site improved the correlation between the calculated and experimental ΔΔ*G*s of weak inhibitors. Although the performance of model A was acceptable also for the weakest inhibitors of the series in the lower part of Figure 5, in certain cases (**2**, **23**, and **18**), model B performed better and the additional exploration of alternative binding modes suggested by automated docking further improved the performance of the model for **6**, **13**, **17**, **19**, **20**, **29**, and **30**.

As explained above, we were able to improve considerably the correlation with the experimental binding free energies of the weaker inhibitors by lowering the strength of the Lewis base that coordinates both the Mn ions (from phenolic oxygen to carboxylate oxygen or amide oxygen) and simultaneously obtaining a non-optimal coordination of the metals (Figure 4a), in line with the hypothesis that weak inhibitors coordinate the metal centers through weak basic atoms.

Interestingly, using a minimal model of the binding site (model A) we were able to identify 60% of the 10 inhibitors with the highest affinity for the target. The results obtained with model B, without considering binding poses obtained through automated docking, could identify 50% of the top 10 inhibitors, while the introduction of docking poses improved this statistic to 70%.

The calculated Pearson coefficients on the whole dataset are 0.5 for model A, 0.4 for model B, and 0.6 for model B when alternative docking poses are considered for compounds **6**, **13**, **15**–**17**, **19**–**21**, **29**, and **30** (cfr. Combining DFT with Automated Docking).

## Conclusions

In this study, we aimed to develop a QM method to understand the SAR and ultimately predict the effect of the variations on the affinity of metalloenzyme inhibitors. Thanks to the explicit treatment of electrons, DFT has the ability to produce accurate descriptions of the interaction between proteins and ligands, allowing for rigorous predictions of binding free energies. We tested our DFT method on a set of fragment-like molecules with varying electronic properties, coordinating the two Mn^2+^ ions of the influenza RNA polymerase PA_N_ endonuclease through a donor triad. We could determine the main contributions to the binding free energies of the inhibitors by comparing the results obtained with two models of the binding pocket with different sizes. Initially, we aligned the dataset to a reduced model of the binding pocket, based on the available X-ray crystallography structures of four inhibitors, hence assuming that all the inhibitors shared a similar binding pose. After determining the relative spin of the Mn^2+^ ions in the ground state, we optimized the geometries of each inhibitor in both bound and free states in gas phase, and thereafter we calculated the energies of both states with water as the implicit solvent. The coordination of the metals resulted to be the main contribution to the relative binding free energy for the majority of the best inhibitors of the series. The ΔΔ*G* predictions for the rest of the dataset were qualitatively correct, except for compounds characterized by heterocycles and/or coordinating the metal centers through nitrogen-containing functional groups.

We tested a bigger model of the binding site to assess the effect of an additional interaction between the ligands and Lys134 on the correlation between our calculations and the experiments.

We noticed that certain inhibitors with low affinity for PA_N_ endonuclease benefitted from the formation of a hydrogen bond with Lys134, which balances the weaker binding to the metal centers typical of weak inhibitors. Additionally, the introduction of binding poses derived by automated docking improved the correlation for the entire dataset. In particular, we observed a marked improvement for molecules that can coordinate the Mn^2+^ ions in multiple ways. A less effective but still plausible, given the distinct structure of the inhibitors, coordination of both ions was beneficial for the predictions of binding free energies of compounds that can coordinate the metal centers through amide groups. This is in agreement with the observation of Credille et al. that low-affinity inhibitors may coordinate the metals with weak Lewis bases.^12^

By introducing automated docking poses, not only could we observe the influence of the identity of the donor atom in metal coordination but also how deviations from the transition state geometry affect the prediction of binding free energies. Although we cannot rule out that compounds with similar cores bind in distinct ways in certain instances, another factor that affects the accuracy of the binding free energy prediction is the suitability of the binding pocket model. It is possible that the ligands interact with residues that our model does not account for. Extending the model of the binding site may improve the predictions but the risk is to increase the computational cost, introduce artifacts, and lose the contribution of the metal coordination, which makes up for the greatest part of the binding free energy. Other factors that generally contribute to the binding free energy of metalloenzyme inhibitors, such as entropy or dynamic effects, were not considered due to the nature of the computational methods employed in this study. However, we were able to rank inhibitors of similar size and with different electronic properties, for which the enthalpic factor is considered to be the main relative contribution to the binding free energy. Our aim was to understand the SAR, focusing on the assessment of the interactions of a series of inhibitors with metal centers and their first shell of coordinating residues, and ultimately contribute to the design of new and improved MBGs. In fact, the DFT-based method described herein, integrated with automated docking, enabled the identification of up to 70% of the top 10 inhibitors in terms of affinity.

In conclusion, we showed that it is possible to predict the relative binding free energies of a series of fragment-like compounds that target a metalloenzyme through a hybrid DFT/docking method using a reduced model of the binding site. Our aim was to model a system where the short-range interactions are prevalent and critical for binding. The PA_N_ endonuclease is a perfect example of such targets, as the main contribution to the binding energy of the inhibitors appears to originate from the coordination of the metal ions of the binding site by the metal binding group of the ligands.^12^ Detailed information on the binding mode of ligands similar to those that are to be tested is preferred, e.g., from crystal structures and/or SAR of the system. Automated docking may help guessing the binding modes but also assessing the features of donor groups that coordinate the metal center(s) of metalloenzymes more efficiently, to guide further design. Overall, the computational method presented herein is able to reproduce the experimental relative binding free energies of a set of inhibitors to the PA_N_ endonuclease. The level of theory is adequate in reflecting the electronic effects of the decorations of the aromatic rings on the coordination of the metal centers of the protein. In addition to being able to discriminate between strong and weak inhibitors, this method is particularly reliable for the prediction of the binding energies of best inhibitors of the dataset. Moreover, it is a tool that facilitates the understanding of the SAR of metalloenzyme inhibitors and the scoring of compounds selected through virtual screening in an early stage of a drug discovery project, even in the absence of experimental data (structural and/or binding affinities), with a limited computational cost.

## Methods

The compound dataset, taken from Credille et al., is reported in Table 1.^12^ Four crystal structures of influenza RNA polymerase PA_N_ endonuclease (PA_N_) in complex with compounds **1**, **2**, **3**, and **28** (PDB IDs: 6DZQ, 6DCY, 6DCZ, and 6E0Q, respectively) were examined to build a model of the binding site for DFT calculations. The hydrogen atoms were added, and the protonation states of the titratable sidechains were assigned with Maestro Protein Preparation Wizard.^63^ The crystal structure with the best resolution, 6DCY, was chosen to build two reduced models of the binding site, including the two Mn^2+^ ions, two water molecules coordinating the Mn1 ion, and all residues directly coordinating the Mn^2+^ ions, i.e., the sidechains of His41 (protonated at the N_δ_) and Asp108 (negatively charged), truncated at the C_β_, the sidechains of Glu80 and Glu119 (both negatively charged), truncated at the C_γ_, and the backbones of Ile120 and Gly121 (model A, Figure 1a). The second model (model B) additionally included the sidechain of Lys134 (positively charged) truncated at the C_ε_ (Figure 1b). The truncated residues were capped with hydrogen atoms. The co-crystallized ligand **2**, in particular, was chosen as reference for the manual docking of compounds **4**–**26**, **29**, and **30**. The binding poses of compounds **1**, **3**, and **28** were extracted from the respective crystal structures (PDB IDs: 6DZQ, 6DCZ, and 6E0Q, respectively), while the co-crystallized compound **28** was used as reference for the manual docking of compound **27**.

### Density Functional Theory

First, the ground state of the system was identified through geometry optimizations and consequent single-point energy (SPE) calculations of model B of the binding site with compound **2** bound. Possible combinations of spin states, derived by different occupations of the d-orbitals of the Mn atoms, were tested (Table S1). The spin combination 5, −5 showed the lowest energy and therefore was adopted throughout the study. This is corroborated by previous studies on dinuclear Mn(II) systems with octahedral coordination that showed antiferromagnetic coupling between the two high-spin ions.^64−66^

All the quantum chemical calculations of the compounds in their free and bound states were performed using the B3LYP-D3 functional.^67^ Each compound in the dataset was optimized in its free and complexed forms in gas phase, employing the 6-31G** basis set (LACVP** for the metal atoms). When needed (see Results and Discussion), optimization of the complexes in water was performed with the same level of theory and the Poisson–Boltzmann finite element solvation model (PBF). Briefly, in this type of calculation the solvent is treated as a dielectric continuum with a cavity for the molecule. The reference energy for solvation is derived from an energy optimization of the structure in gas phase. Later, the solution phase energies were derived from single-point energy calculations performed on the previously optimized structures using the cc-pVTZ(-f) basis set (LACV3P** for the metal atoms) and the Poisson–Boltzmann finite element solvation model (PBF).^68,69^ Considering the water accessibility of the binding site, we used water as the solvent and employed a dielectric constant ε = 80 in our calculations.

The truncation points were fixed during the geometry optimizations, together with the hydrogen cap atoms along the truncated vectors, to represent the strain of the surrounding residues.

All the DFT calculations were performed using Jaguar (Jaguar, version 10.1, Schrodinger, Inc., New York, NY, 2018).^70^

The Δ*G*s were calculated as the difference in energy between a model of the protein–ligand complex and the free ligand in solution. The relative binding free energies (ΔΔ*G*) were calculated using the binding free energy (Δ*G*) of compound **1** as reference.
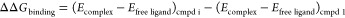

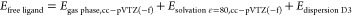

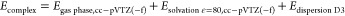


### Docking

GOLD (version 5.2.2)^71^ was used to explore additional binding poses for the compounds included in this study. GOLD uses a genetic algorithm to provide optimized docking poses. A maximum of 50 poses for each possible charge state of the compounds in the dataset were generated and scored with the ChemScore scoring function.^72,73^ The coordination geometry of the two Mn ions was recognized by GOLD as octahedral after superimposition of coordination templates on the protein coordinating atoms. The binding pocket was defined by all the residues of 6DCY within 10 Å of the co-crystallized ligand. The water molecules in the binding site defined as above were all retained and toggled on and off by GOLD during the docking runs. The protein structure was considered rigid.

## Data Availability

All the starting PDB files were downloaded from the RCSB Protein Data Bank (https://www.rcsb.org/). The dataset was derived from Credille et al.^12^ Any computational data generated and analyzed for this study that are not included in this article and the Supporting Information are available from the authors upon request. The Schrödinger software suite is commercial software that can be trialed for free on request to the vendor (https://www.schrodinger.com/). The GOLD docking suite can be trialed on request to CCDC Software Ltd. (https://www.ccdc.cam.ac.uk/solutions/csd-discovery/components/gold/).

## Supporting Information Available

Relative energies for possible spin combinations of the two Mn^2+^; p*K*_a_ values of titratable groups of the compounds of the dataset and description of methodology; relative binding energies of docking poses for selected inhibitors tested with model A; docking binding pose of compound **16**; comparison of the relative binding free energies of the co-crystallized inhibitors obtained with larger binding site models (PDF)
Coordinate files (*xyz*) of the optimized structures (ligands in their free state and ligands bound to models A–F) (ZIP)

## Abbreviations

DFT: density functional theory
FBDD: fragment-based drug discovery
FEP: free energy perturbation
MBG: metal binding group
MC: Monte Carlo
MD: molecular dynamics
QM: quantum mechanics
SAR: structure–activity relationship
SPE: single-point energy

## References

[ref1] Ma X.; Ezzeldin H. H.; Diasio R. B. Histone Deacetylase Inhibitors: Current Status and Overview of Recent Clinical Trials. Drugs 2009, 69, 1911–1934. 10.2165/11315680-000000000-00000. 19747008

[ref2] Fisher J. F.; Mobashery S. Recent Advances in MMP Inhibitor Design. Cancer Metastasis Rev. 2006, 25, 115–136. 10.1007/s10555-006-7894-9. 16680577

[ref3] Dive V.; Chang C.-F.; Yiotakis A.; Sturrock E. D. Inhibition of Zinc Metallopeptidases in Cardiovascular Disease--from Unity to Trinity, or Duality?. Curr. Pharm. Des. 2009, 15, 3606–3621. 10.2174/138161209789271889. 19925415

[ref4] Dunkel P.; Gelain A.; Barlocco D.; Haider N.; Gyires K.; Sperlágh B.; Magyar K.; Maccioni E.; Fadda A.; Mátyus P. Semicarbazide-Sensitive Amine Oxidase/Vascular Adhesion Protein 1: Recent Developments Concerning Substrates and Inhibitors of a Promising Therapeutic Target. Curr. Med. Chem. 2008, 15, 1827–1839. 10.2174/092986708785133022. 18691041

[ref5] Mai A.; Rotili D.; Valente S.; Kazantsev A. G. Histone Deacetylase Inhibitors and Neurodegenerative Disorders: Holding the Promise. Curr. Pharm. Des. 2009, 15, 3940–3957. 10.2174/138161209789649349. 19751207

[ref6] Burnett J. C.; Henchal E. A.; Schmaljohn A. L.; Bavari S. The Evolving Field of Biodefence: Therapeutic Developments and Diagnostics. Nat. Rev. Drug Discovery 2005, 4, 281–296. 10.1038/nrd1694. 15803193 PMC7096857

[ref7] Hernick M.; Fierke C. A. Zinc Hydrolases: The Mechanisms of Zinc-Dependent Deacetylases. Arch. Biochem. Biophys. 2005, 433, 71–84. 10.1016/j.abb.2004.08.006. 15581567

[ref8] Villain-Guillot P.; Bastide L.; Gualtieri M.; Leonetti J.-P. Progress in Targeting Bacterial Transcription. Drug Discovery Today 2007, 12, 200–208. 10.1016/j.drudis.2007.01.005. 17331884

[ref9] Dubey S.; Satyanarayana Y. D.; Lavania H. Development of Integrase Inhibitors for Treatment of AIDS: An Overview. Eur. J. Med. Chem. 2007, 42, 1159–1168. 10.1016/j.ejmech.2007.01.024. 17367896

[ref10] White R. J.; Margolis P. S.; Trias J.; Yuan Z. Targeting Metalloenzymes: A Strategy That Works. Curr. Opin. Pharmacol. 2003, 3, 502–507. 10.1016/s1471-4892(03)00115-2. 14559095

[ref11] Vaughan M. D.; Sampson P. B.; Honek J. F. Methionine in and out of Proteins: Targets for Drug Design. Curr. Med. Chem. 2002, 9, 385–409. 10.2174/0929867023371102. 11860363

[ref12] Credille C. V.; Dick B. L.; Morrison C. N.; Stokes R. W.; Adamek R. N.; Wu N. C.; Wilson I. A.; Cohen S. M. Structure–Activity Relationships in Metal-Binding Pharmacophores for Influenza Endonuclease. J. Med. Chem. 2018, 61, 10206–10217. 10.1021/acs.jmedchem.8b01363. 30351002 PMC6249039

[ref13] Chen A. Y.; Adamek R. N.; Dick B. L.; Credille C. V.; Morrison C. N.; Cohen S. M. Targeting Metalloenzymes for Therapeutic Intervention. Chem. Rev. 2019, 119, 1323–1455. 10.1021/acs.chemrev.8b00201. 30192523 PMC6405328

[ref14] Day J. A.; Cohen S. M. Investigating the Selectivity of Metalloenzyme Inhibitors. J. Med. Chem. 2013, 56, 7997–8007. 10.1021/jm401053m. 24074025 PMC3880651

[ref15] Martin D. P.; Puerta D. T.; Cohen S. M. Metalloprotein Inhibitors. In Ligand Design in Medicinal Inorganic Chemistry; Storr T., Ed.; John Wiley & Sons, Ltd: Chichester, UK, 2014; pp. 375–403, 10.1002/9781118697191.ch14.

[ref16] Coussens L. M.; Fingleton B.; Matrisian L. M. Matrix Metalloproteinase Inhibitors and Cancer: Trials and Tribulations. Science 2002, 295, 2387–2392. 10.1126/science.1067100. 11923519

[ref17] Frey R. R.; Wada C. K.; Garland R. B.; Curtin M. L.; Michaelides M. R.; Li J.; Pease L. J.; Glaser K. B.; Marcotte P. A.; Bouska J. J.; Murphy S. S.; Davidsen S. K. Trifluoromethyl Ketones as Inhibitors of Histone Deacetylase. Bioorg. Med. Chem. Lett. 2002, 12, 3443–3447. 10.1016/s0960-894x(02)00754-0. 12419380

[ref18] Reiter L. A.; Robinson R. P.; McClure K. F.; Jones C. S.; Reese M. R.; Mitchell P. G.; Otterness I. G.; Bliven M. L.; Liras J.; Cortina S. R.; Donahue K. M.; Eskra J. D.; Griffiths R. J.; Lame M. E.; Lopez-Anaya A.; Martinelli G. J.; McGahee S. M.; Yocum S. A.; Lopresti-Morrow L. L.; Tobiassen L. M.; Vaughn-Bowser M. L. Pyran-Containing Sulfonamide Hydroxamic Acids: Potent MMP Inhibitors That Spare MMP-1. Bioorg. Med. Chem. Lett. 2004, 14, 3389–3395. 10.1016/j.bmcl.2004.04.083. 15177439

[ref19] Lin J. H.; Lu A. Y. Role of Pharmacokinetics and Metabolism in Drug Discovery and Development. Pharmacol. Rev. 1997, 49, 403–449. 9443165

[ref20] Edmonds S.; Gibb A.; Sim E. Effect of Thiol Compounds on Human Complement Component C4. Biochem. J. 1993, 289, 801–805. 10.1042/bj2890801. 8435078 PMC1132247

[ref21] Credille C. V.; Chen Y.; Cohen S. M. Fragment-Based Identification of Influenza Endonuclease Inhibitors. J. Med. Chem. 2016, 59, 6444–6454. 10.1021/acs.jmedchem.6b00628. 27291165 PMC4948595

[ref22] Congreve M.; Chessari G.; Tisi D.; Woodhead A. J. Recent Developments in Fragment-Based Drug Discovery. J. Med. Chem. 2008, 51, 3661–3680. 10.1021/jm8000373. 18457385

[ref23] Jacobsen J. A.; Fullagar J. L.; Miller M. T.; Cohen S. M. Identifying Chelators for Metalloprotein Inhibitors Using a Fragment-Based Approach. J. Med. Chem. 2011, 54, 591–602. 10.1021/jm101266s. 21189019 PMC3024453

[ref24] Gohlke H.; Klebe G. Approaches to the Description and Prediction of the Binding Affinity of Small-Molecule Ligands to Macromolecular Receptors. Angew. Chem., Int. Ed. 2002, 41, 2644–2676. 10.1002/1521-3773(20020802)41:15<2644::AID-ANIE2644>3.0.CO;2-O. 12203463

[ref25] Ryde U.; Söderhjelm P. Ligand-Binding Affinity Estimates Supported by Quantum-Mechanical Methods. Chem. Rev. 2016, 116, 5520–5566. 10.1021/acs.chemrev.5b00630. 27077817

[ref26] Palermo G.; Spinello A.; Saha A.; Magistrato A. Frontiers of Metal-Coordinating Drug Design. Expert Opin. Drug Discovery 2021, 16, 497–511. 10.1080/17460441.2021.1851188. PMC8058448 33874825

[ref27] Warren G. L.; Andrews C. W.; Capelli A.-M.; Clarke B.; LaLonde J.; Lambert M. H.; Lindvall M.; Nevins N.; Semus S. F.; Senger S.; Tedesco G.; Wall I. D.; Woolven J. M.; Peishoff C. E.; Head M. S. A Critical Assessment of Docking Programs and Scoring Functions. J. Med. Chem. 2006, 49, 5912–5931. 10.1021/jm050362n. 17004707

[ref28] Karges J.; Stokes R. W.; Cohen S. M. Computational Prediction of the Binding Pose of Metal-Binding Pharmacophores. ACS Med. Chem. Lett. 2022, 13, 428–435. 10.1021/acsmedchemlett.1c00584. 35300086 PMC8919381

[ref29] Boresch S.; Tettinger F.; Leitgeb M.; Karplus M. Absolute Binding Free Energies: A Quantitative Approach for Their Calculation. J. Phys. Chem. B 2003, 107, 9535–9551. 10.1021/jp0217839.

[ref30] Lim N. M.; Wang L.; Abel R.; Mobley D. L. Sensitivity in Binding Free Energies Due to Protein Reorganization. J. Chem. Theory Comput. 2016, 12, 4620–4631. 10.1021/acs.jctc.6b00532. 27462935 PMC5021633

[ref31] Boresch S.; Karplus M. The Role of Bonded Terms in Free Energy Simulations: 1. Theoretical Analysis. J. Phys. Chem. A 1999, 103, 103–118. 10.1021/jp981628n.

[ref32] Wang L.; Deng Y.; Wu Y.; Kim B.; LeBard D. N.; Wandschneider D.; Beachy M.; Friesner R. A.; Abel R. Accurate Modeling of Scaffold Hopping Transformations in Drug Discovery. J. Chem. Theory Comput. 2017, 13, 42–54. 10.1021/acs.jctc.6b00991. 27933808

[ref33] Hansen N.; van Gunsteren W. F. Practical Aspects of Free-Energy Calculations: A Review. J. Chem. Theory Comput. 2014, 10, 2632–2647. 10.1021/ct500161f. 26586503

[ref34] Kumbhar S.; Fischer F. D.; Waller M. P. Assessment of Weak Intermolecular Interactions Across QM/MM Noncovalent Boundaries. J. Chem. Inf. Model. 2012, 52, 93–98. 10.1021/ci200406s. 22185219

[ref35] Sherrill C. D.; Sumpter B. G.; Sinnokrot M. O.; Marshall M. S.; Hohenstein E. G.; Walker R. C.; Gould I. R. Assessment of Standard Force Field Models against High-Quality *Ab Initio* Potential Curves for Prototypes of π-π, CH/π, and SH/π Interactions. J. Comput. Chem. 2009, 30, 2187–2193. 10.1002/jcc.21226. 19242959

[ref36] Parker T. M.; Sherrill C. D. Assessment of Empirical Models versus High-Accuracy Ab Initio Methods for Nucleobase Stacking: Evaluating the Importance of Charge Penetration. J. Chem. Theory Comput. 2015, 11, 4197–4204. 10.1021/acs.jctc.5b00588. 26575915

[ref37] Ehrlich S.; Göller A. H.; Grimme S. Towards Full Quantum-Mechanics-Based Protein-Ligand Binding Affinities. ChemPhysChem 2017, 18, 898–905. 10.1002/cphc.201700082. 28133881

[ref38] Mucs D.; Bryce R. A. The Application of Quantum Mechanics in Structure-Based Drug Design. Expert Opin. Drug Discovery 2013, 8, 263–276. 10.1517/17460441.2013.752812. 23289945

[ref39] Raha K.; Peters M. B.; Wang B.; Yu N.; Wollacott A. M.; Westerhoff L. M.; Merz K. M. The Role of Quantum Mechanics in Structure-Based Drug Design. Drug Discovery Today 2007, 12, 725–731. 10.1016/j.drudis.2007.07.006. 17826685

[ref40] Roos K.; Viklund J.; Meuller J.; Kaspersson K.; Svensson M. Potency Prediction of β-Secretase (BACE-1) Inhibitors Using Density Functional Methods. J. Chem. Inf. Model. 2014, 54, 818–825. 10.1021/ci400374z. 24456077

[ref41] Siegbahn P. E. M.; Himo F. Recent Developments of the Quantum Chemical Cluster Approach for Modeling Enzyme Reactions. JBIC J. Biol. Inorg. Chem. 2009, 14, 643–651. 10.1007/s00775-009-0511-y. 19437047

[ref42] Blomberg M. R. A.; Borowski T.; Himo F.; Liao R.-Z.; Siegbahn P. E. M. Quantum Chemical Studies of Mechanisms for Metalloenzymes. Chem. Rev. 2014, 114, 3601–3658. 10.1021/cr400388t. 24410477

[ref43] Nichol K. L.; Treanor J. J. Vaccines for Seasonal and Pandemic Influenza. J. Infect. Dis. 2006, 194, S111–S118. 10.1086/507544. 17163383

[ref44] Monod A.; Swale C.; Tarus B.; Tissot A.; Delmas B.; Ruigrok R. W.; Crépin T.; Slama-Schwok A. Learning from Structure-Based Drug Design and New Antivirals Targeting the Ribonucleoprotein Complex for the Treatment of Influenza. Expert Opin. Drug Discovery 2015, 10, 345–371. 10.1517/17460441.2015.1019859. 25792362

[ref45] Drake J. W. Rates of Spontaneous Mutation among RNA Viruses. Proc. Natl. Acad. Sci. 1993, 90, 4171–4175. 10.1073/pnas.90.9.4171. 8387212 PMC46468

[ref46] Watanabe T.; Kawaoka Y. Influenza Virus–Host Interactomes as a Basis for Antiviral Drug Development. Curr. Opin. Virol. 2015, 14, 71–78. 10.1016/j.coviro.2015.08.008. 26364134 PMC5380926

[ref47] Dias A.; Bouvier D.; Crépin T.; McCarthy A. A.; Hart D. J.; Baudin F.; Cusack S.; Ruigrok R. W. H. The Cap-Snatching Endonuclease of Influenza Virus Polymerase Resides in the PA Subunit. Nature 2009, 458, 914–918. 10.1038/nature07745. 19194459

[ref48] Yuan P.; Bartlam M.; Lou Z.; Chen S.; Zhou J.; He X.; Lv Z.; Ge R.; Li X.; Deng T.; Fodor E.; Rao Z.; Liu Y. Crystal Structure of an Avian Influenza Polymerase PAN Reveals an Endonuclease Active Site. Nature 2009, 458, 909–913. 10.1038/nature07720. 19194458

[ref49] Ortín J.; Martín-Benito J. The RNA Synthesis Machinery of Negative-Stranded RNA Viruses. Virology 2015, 479-480, 532–544. 10.1016/j.virol.2015.03.018. 25824479

[ref50] Moeller A.; Kirchdoerfer R. N.; Potter C. S.; Carragher B.; Wilson I. A. Organization of the Influenza Virus Replication Machinery. Science 2012, 338, 1631–1634. 10.1126/science.1227270. 23180774 PMC3578580

[ref51] Kowalinski E.; Zubieta C.; Wolkerstorfer A.; Szolar O. H. J.; Ruigrok R. W. H.; Cusack S. Structural Analysis of Specific Metal Chelating Inhibitor Binding to the Endonuclease Domain of Influenza PH1N1 (2009) Polymerase. PLoS Pathog. 2012, 8, e1002831 10.1371/journal.ppat.1002831. 22876177 PMC3410856

[ref52] DuBois R. M.; Slavish P. J.; Baughman B. M.; Yun M.-K.; Bao J.; Webby R. J.; Webb T. R.; White S. W. Structural and Biochemical Basis for Development of Influenza Virus Inhibitors Targeting the PA Endonuclease. PLoS Pathog. 2012, 8, e1002830 10.1371/journal.ppat.1002830. 22876176 PMC3410894

[ref53] Nakazawa M.; Kadowaki S.; Watanabe I.; Kadowaki Y.; Takei M.; Fukuda H. PA Subunit of RNA Polymerase as a Promising Target for Anti-Influenza Virus Agents. Antiviral Res. 2008, 78, 194–201. 10.1016/j.antiviral.2007.12.010. 18258312

[ref54] Sagong H. Y.; Bauman J. D.; Patel D.; Das K.; Arnold E.; LaVoie E. J. Phenyl Substituted 4-Hydroxypyridazin-3(2 *H* )-Ones and 5-Hydroxypyrimidin-4(3 *H* )-Ones: Inhibitors of Influenza A Endonuclease. J. Med. Chem. 2014, 57, 8086–8098. 10.1021/jm500958x. 25225968 PMC4191602

[ref55] Bauman J. D.; Patel D.; Baker S. F.; Vijayan R. S. K.; Xiang A.; Parhi A. K.; Martínez-Sobrido L.; LaVoie E. J.; Das K.; Arnold E. Crystallographic Fragment Screening and Structure-Based Optimization Yields a New Class of Influenza Endonuclease Inhibitors. ACS Chem. Biol. 2013, 8, 2501–2508. 10.1021/cb400400j. 23978130 PMC3928712

[ref56] Zhao C.; Lou Z.; Guo Y.; Ma M.; Chen Y.; Liang S.; Zhang L.; Chen S.; Li X.; Liu Y.; Bartlam M.; Rao Z. Nucleoside Monophosphate Complex Structures of the Endonuclease Domain from the Influenza Virus Polymerase PA Subunit Reveal the Substrate Binding Site inside the Catalytic Center. J. Virol. 2009, 83, 9024–9030. 10.1128/JVI.00911-09. 19587036 PMC2738217

[ref57] Tefsen B.; Lu G.; Zhu Y.; Haywood J.; Zhao L.; Deng T.; Qi J.; Gao G. F. The N-Terminal Domain of PA from Bat-Derived Influenza-Like Virus H17N10 Has Endonuclease Activity. J. Virol. 2014, 88, 1935–1941. 10.1128/JVI.03270-13. 24284327 PMC3911528

[ref58] Boivin S.; Cusack S.; Ruigrok R. W. H.; Hart D. J. Influenza A Virus Polymerase: Structural Insights into Replication and Host Adaptation Mechanisms. J. Biol. Chem. 2010, 285, 28411–28417. 10.1074/jbc.R110.117531. 20538599 PMC2937865

[ref59] Crépin T.; Dias A.; Palencia A.; Swale C.; Cusack S.; Ruigrok R. W. H. Mutational and Metal Binding Analysis of the Endonuclease Domain of the Influenza Virus Polymerase PA Subunit. J. Virol. 2010, 84, 9096–9104. 10.1128/JVI.00995-10. 20592097 PMC2937609

[ref60] Noble E.; Cox A.; Deval J.; Kim B. Endonuclease Substrate Selectivity Characterized with Full-Length PA of Influenza A Virus Polymerase. Virology 2012, 433, 27–34. 10.1016/j.virol.2012.07.008. 22841552 PMC3647620

[ref61] Omoto S.; Speranzini V.; Hashimoto T.; Noshi T.; Yamaguchi H.; Kawai M.; Kawaguchi K.; Uehara T.; Shishido T.; Naito A.; Cusack S. Characterization of Influenza Virus Variants Induced by Treatment with the Endonuclease Inhibitor Baloxavir Marboxil. Sci. Rep. 2018, 8, 9633 10.1038/s41598-018-27890-4. 29941893 PMC6018108

[ref62] Song M.-S.; Kumar G.; Shadrick W. R.; Zhou W.; Jeevan T.; Li Z.; Slavish P. J.; Fabrizio T. P.; Yoon S.-W.; Webb T. R.; Webby R. J.; White S. W. Identification and Characterization of Influenza Variants Resistant to a Viral Endonuclease Inhibitor. Proc. Natl. Acad. Sci. 2016, 113, 3669–3674. 10.1073/pnas.1519772113. 26976575 PMC4822642

[ref63] Madhavi Sastry G.; Adzhigirey M.; Day T.; Annabhimoju R.; Sherman W. Protein and Ligand Preparation: Parameters, Protocols, and Influence on Virtual Screening Enrichments. J. Comput.-Aided Mol. Des. 2013, 27, 221–234. 10.1007/s10822-013-9644-8. 23579614

[ref64] Howard T.; Telser J.; DeRose V. J. An Electron Paramagnetic Resonance Study of Mn _2_ (H _2_ O)(OAc) _4_ (Tmeda) _2_ (Tmeda = *N* , *N* , *N* ‘, *N* ‘-Tetramethylethylenediamine): A Model for Dinuclear Manganese Enzyme Active Sites. Inorg. Chem. 2000, 39, 3379–3385. 10.1021/ic0000247. 11196878

[ref65] Yu S. B.; Lippard S. J.; Shweky I.; Bino A. Dinuclear Manganese(II) Complexes with Water and Carboxylate Bridges. Inorg. Chem. 1992, 31, 3502–3504. 10.1021/ic00043a004.

[ref66] Golombek A. P.; Hendrich M. P. Quantitative Analysis of Dinuclear Manganese(II) EPR Spectra. J. Magn. Reson. 2003, 165, 33–48. 10.1016/j.jmr.2003.07.001. 14568515

[ref67] Grimme S.; Antony J.; Ehrlich S.; Krieg H. A Consistent and Accurate *Ab Initio* Parametrization of Density Functional Dispersion Correction (DFT-D) for the 94 Elements H-Pu. J. Chem. Phys. 2010, 132, 154104 10.1063/1.3382344. 20423165

[ref68] Tannor D. J.; Marten B.; Murphy R.; Friesner R. A.; Sitkoff D.; Nicholls A.; Honig B.; Ringnalda M.; Goddard W. A. Accurate First Principles Calculation of Molecular Charge Distributions and Solvation Energies from Ab Initio Quantum Mechanics and Continuum Dielectric Theory. J. Am. Chem. Soc. 1994, 116, 11875–11882. 10.1021/ja00105a030.

[ref69] Marten B.; Kim K.; Cortis C.; Friesner R. A.; Murphy R. B.; Ringnalda M. N.; Sitkoff D.; Honig B. New Model for Calculation of Solvation Free Energies: Correction of Self-Consistent Reaction Field Continuum Dielectric Theory for Short-Range Hydrogen-Bonding Effects. J. Phys. Chem. 1996, 100, 11775–11788. 10.1021/jp953087x.

[ref70] Bochevarov A. D.; Harder E.; Hughes T. F.; Greenwood J. R.; Braden D. A.; Philipp D. M.; Rinaldo D.; Halls M. D.; Zhang J.; Friesner R. A. Jaguar: A High-performance Quantum Chemistry Software Program with Strengths in Life and Materials Sciences. Int. J. Quantum Chem. 2013, 113, 2110–2142. 10.1002/qua.24481.

[ref71] Verdonk M. L.; Cole J. C.; Hartshorn M. J.; Murray C. W.; Taylor R. D. Improved Protein-Ligand Docking Using GOLD. Proteins: Struct., Funct., Bioinf. 2003, 52, 609–623. 10.1002/prot.10465. 12910460

[ref72] Eldridge M. D.; Murray C. W.; Auton T. R.; Paolini G. V.; Mee R. P. Empirical Scoring Functions: I. The Development of a Fast Empirical Scoring Function to Estimate the Binding Affinity of Ligands in Receptor Complexes. J. Comput.-Aided Mol. Des. 1997, 11, 425–445. 10.1023/A:1007996124545. 9385547

[ref73] Baxter C. A.; Murray C. W.; Clark D. E.; Westhead D. R.; Eldridge M. D. Flexible Docking Using Tabu Search and an Empirical Estimate of Binding Affinity. Proteins: Struct., Funct., Genet. 1998, 33, 367–382. 10.1002/(SICI)1097-0134(19981115)33:3<367::AID-PROT6>3.0.CO;2-W. 9829696

