# Supplementary material for: Understanding the Structure–Activity Relationship through Density Functional Theory: A Simple Method Predicts Relative Binding Free Energies of Metalloenzyme Fragment-like Inhibitors
Source: ACS Omega. 2023 Jun 6;8(24):21438–49. doi: 10.1021/acsomega.2c08156 (PMC10285960; doi:10.1021/acsomega.2c08156)
Supplement: Supplementary file 1 — ao2c08156_si_001.pdf [file ao2c08156_si_001.pdf]

Understanding SAR through DFT: a simple method predicts relative binding free energies of metalloenzyme fragment-like inhibitors

Silvana Vasile[a], Katarina Roos[a]*

[a]Department of Cell and Molecular Biology, Uppsala University, Uppsala, Sweden

Supporting Information

Relative energies for possible spin combinations of the two Mn^{2+} ____ *2*

Results of pKa calculations on the dataset ____ *3*

pKa calculations ____ 4

Relative binding free energies of alternative binding modes for selected inhibitors tested in Model A ____ *5*

Comparison of relative binding free energies of the automated docking binding modes considered for selected inhibitors ____ *6*

Docking binding pose of compound 16 ____ *7*

Comparison of the relative binding free energies of the co-crystallised inhibitors obtained with larger binding site models ____ *8*

References ____ *10*

Relative energies for possible spin combinations of the two Mn^{2+}

Spin state	Relative E (kcal/mol)
5, -5	0
-3, 5	36.6
5, -3	39.3
5, -1	42.5
-3, 1	86.9

Table S1. Calculated relative binding energies of compound **2** in Model B with different combinations of spin states for the Mn centers. All the antiferromagnetic combinations were tested. The combinations not reported (3, -3; -1,5; -1,3; 1,-1) all resulted in the 5, -5 state after geometry optimization

Results of pKa calculations on the dataset

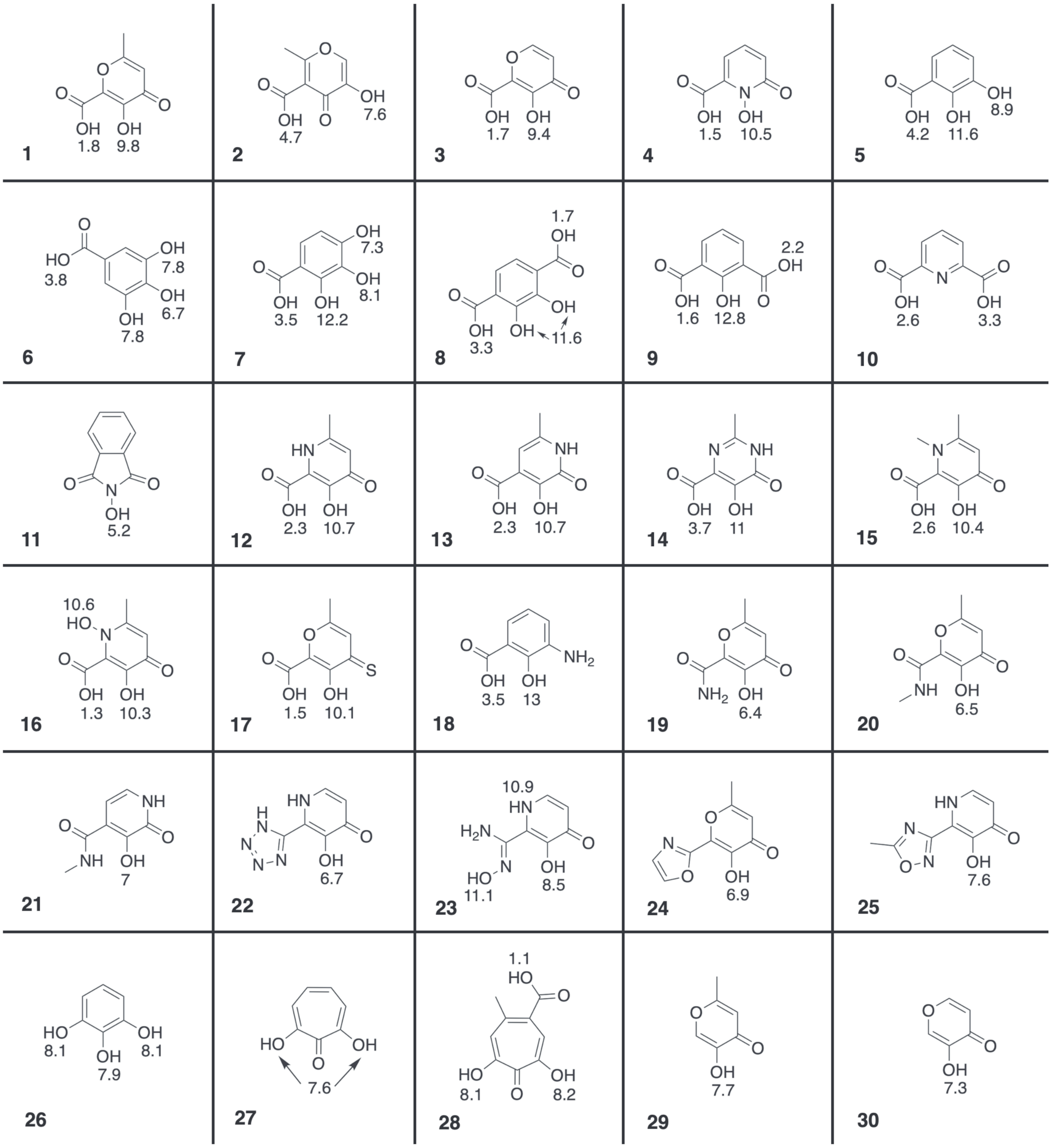

Table S2. pKas of titratable groups of each compound of the dataset.

pKa calculations

The pKas of all protonation sites for each compound in the dataset were calculated in water using Schrödinger's Jaguar pKa prediction module.[1] The protonation sites were manually picked for each inhibitor. The pKa calculations were performed on each site, preceded by conformational searches on the protonated and deprotonated forms of the molecule with MacroModel (MacroModel, Schrödinger, LLC, New York, NY, 2019), according to the module's protocol. The charge state of the molecule was determined at pH = 8 (the pH at which the experimental assays were performed in the original study by Credille et al.).[2] For molecules with multiple protonation sites with similar pKas, the pKa calculations were repeated deprotonating one site at a time. Finally, the total charge state(s) of each inhibitor was determined.

Relative binding free energies of alternative binding modes for selected inhibitors tested in Model A

Compound	$\Delta\Delta G_{calc}$ (kcal/mol)	Compound	$\Delta\Delta G_{calc}$ (kcal/mol)
**6** 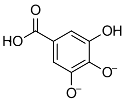	9.5	**19** 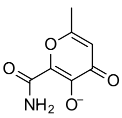	13.8
**13** 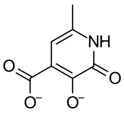	11.1	**20** 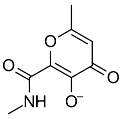	16.8
**15** 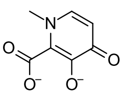	9.9	**21** 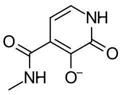	14.0
**16** 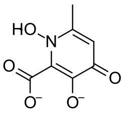	-0.8	**29** 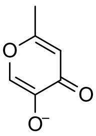	4.7
**17** 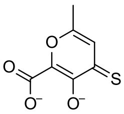	13.7	**30** 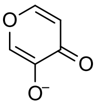	9.0

Table S3. Calculated relative binding free energies of selected PA_N endonuclease inhibitors in Model A, tested in alternative binding modes derived from automated docking.

Comparison of relative binding free energies of the automated docking binding modes considered for selected inhibitors

Compound	$\Delta\Delta G_{exp}$ (kcal/mol)	$\Delta\Delta G_{docking}$ (kcal/mol)	$\Delta\Delta G_{calc}$ Model B (kcal/mol)	Compound	$\Delta\Delta G_{exp}$ (kcal/mol)	$\Delta\Delta G_{docking}$ (kcal/mol)	$\Delta\Delta G_{calc}$ Model B (kcal/mol)
**6** 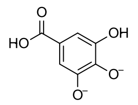	0	0	0	**20** 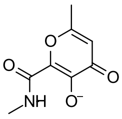	0	-4.2	-0.5
**13** 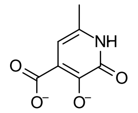	-1.4	-9.1	-1.8	**21** 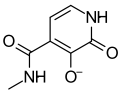	1.5	6.1	-1.5
**15** 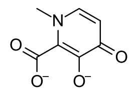	0	-16.1	-4	**29** 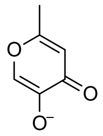	0.9	-4.6	-0.9
**17** 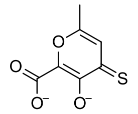	-1.4	-13.4	-1.3	**30** 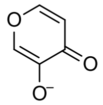	1.1	-6.7	-0.3
**19** 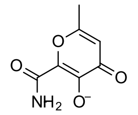	-0.3	0.6	-1.7				

Table S4. Comparison of the relative binding free energies (reference: ΔΔG of compound **6**) of selected PA_N endonuclease inhibitors, tested in alternative binding modes derived from automated docking.

Docking binding pose of compound 16

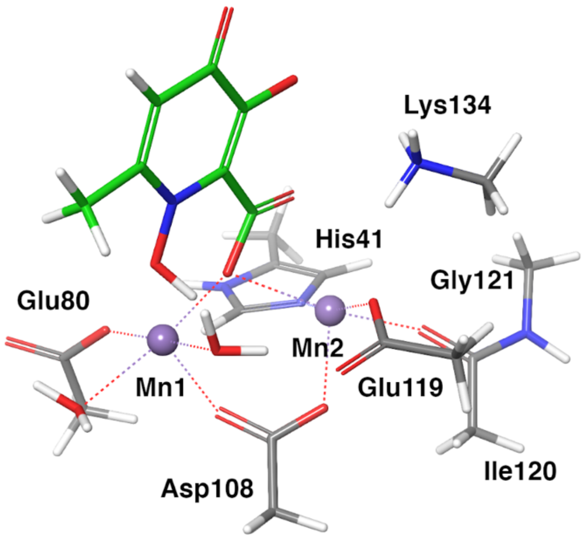

Figure S1. Binding pose of compound **16** identified via docking as most favored. The inhibitor (green sticks) coordinates the two manganese ions (violet spheres) with its carboxylate group.

Comparison of the relative binding free energies of the co-crystallised inhibitors obtained with larger binding site models

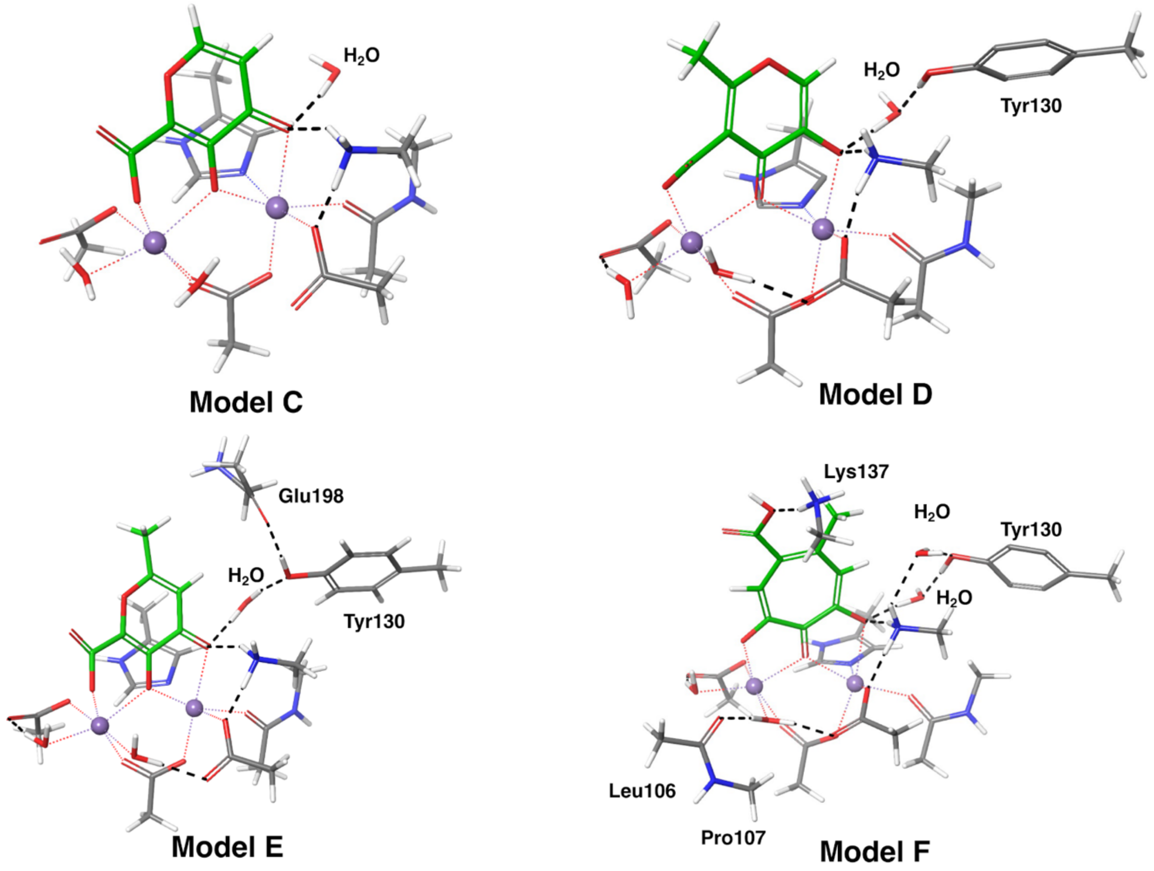

Figure S2. Four binding site models including additional residues in complex with co-crystallised inhibitors (green sticks). **Model C** includes the same atoms as Model B and an additional conserved water molecule interacting with the ligand (compound **1**); **Model D** (complex with compound **2**) includes the conserved water molecule introduced in Model C and the sidechain of Tyr130 forming a hydrogen bond with it; **Model E** (complex with compound **3**) includes all the atoms of Model D and the sidechain of Glu198 capped with a methyl group; **Model F** (complex with compound **28**) includes all the atoms of Model D and additionally the sidechain of Lys137, a water molecule and the backbones of Leu106 and Pro107.

Compound	$\Delta\Delta G_{exp}$ (kcal/mol)	$\Delta\Delta G_{calc}$ Model A (kcal/mol)	$\Delta\Delta G_{calc}$ Model B (kcal/mol)	$\Delta\Delta G_{calc}$ Model C (kcal/mol)	$\Delta\Delta G_{calc}$ Model D (kcal/mol)	$\Delta\Delta G_{calc}$ Model E (kcal/mol)	$\Delta\Delta G_{calc}$ Model F (kcal/mol)
**1**	0	0	0	0	0	0	0
**2**	2.7	4.0	2.7	-1.9	-0.01	1.3	-1.4
**3**	0.3	0.5	0.6	-0.8	-0.4	2.6	1.4
**28**	-1.0	-1.6	0.1	-7.8	-11.6	-2.5	0.7

Table S5. Comparison of the relative binding free energies (reference: ΔΔG of compound **1**) of the co-crystallised PA_N endonuclease inhibitors **1**, **2**, **3** and **28** obtained with the six binding site models (A-F).

References

(1) Bochevarov, A. D.; Harder, E.; Hughes, T. F.; Greenwood, J. R.; Braden, D. A.; Philipp, D. M.; Rinaldo, D.; Halls, M. D.; Zhang, J.; Friesner, R. A. Jaguar: A High-performance Quantum Chemistry Software Program with Strengths in Life and Materials Sciences. *Int. J. Quantum Chem.* **2013**, *113* (18), 2110–2142. https://doi.org/10.1002/qua.24481.

(2) Credille, C. V.; Dick, B. L.; Morrison, C. N.; Stokes, R. W.; Adamek, R. N.; Wu, N. C.; Wilson, I. A.; Cohen, S. M. Structure–Activity Relationships in Metal-Binding Pharmacophores for Influenza Endonuclease. *J. Med. Chem.* **2018**, *61* (22), 10206–10217. https://doi.org/10.1021/acs.jmedchem.8b01363.
